# Lymphocyte Changes in Severe COVID-19: Delayed Over-Activation of STING?

**DOI:** 10.3389/fimmu.2020.607069

**Published:** 2020-12-01

**Authors:** Jean-Marie Berthelot, Frédéric Lioté, Yves Maugars, Jean Sibilia

**Affiliations:** ^1^Rheumatology Department, Nantes University Hospital, Nantes, France; ^2^Rheumatology Department & Inserm UMR 1132 (centre Viggo Petersen), Hôpital Lariboisière, Université de Paris, Paris, France; ^3^Service de rhumatologie, Hopitaux Universitaires de Strasbourg, RESO: Centre de Reference des Maladies Autoimmunes Systemiques Rares Est Sud-Ouest, Strasbourg, France; ^4^INSERM UMR_S1109, Universite de Strasbourg, Strasbourg, France

**Keywords:** SARS-CoV-2, cGAMP, lymphopenia, interferon, STING, COVID-19, lymphocyte, aspirin

## Abstract

Upon recognition of microbial DNA or self-DNA, the cyclic-GMP-AMP synthase (cGAS) of the host catalyzes the production of the cyclic dinucleotide cGAMP. cGAMP is the main activator of STING, stimulator of interferon genes, leading to interferon synthesis through the STING-TBK1-IRF3 pathway. STING is also a hub for activation of NF-κB and autophagy. The present review details the striking similarities between T and B cell responses in severe coronavirus disease 2019 (COVID-19) and both animal or human models of STING gain of function (SAVI syndromes: STING-associated vasculopathy with onset in infancy). Those similarities may be further clues for a delayed activation of STING in severe COVID-19 patients, due to DNA damages following severe acute respiratory syndrome coronaviruses (SARS-CoV-2) infection and unusual role of STING in SARS-CoV-2 control. In early stages, Th2 differentiation are noticed in both severe COVID-19 and SAVI syndromes; then, CD4+ and CD8+ T cells functional exhaustion/senescent patterns due to TCR hyper-responsiveness are observed. T cell delayed over-responses can contribute to pneumonitis and delayed cytokine secretion with over-production of IL-6. Last, STING over-activation induces progressive CD4+ and CD8+ T lymphopenia in SAVI syndromes, which parallels what is observed in severe COVID-19. ACE2, the main receptor of SARS-CoV-2, is rarely expressed in immune cells, and it has not been yet proven that some human lymphocytes could be infected by SARS-CoV-2 through CD147 or CD26. However, STING, expressed in humans T cells, might be triggered following excessive transfer of cGAMP from infected antigen presenting cells into activated CD4+ and CD8+ T cells lymphocytes. Indeed, those lymphocytes highly express the cGAMP importer SLC19A1. Whereas STING is not expressed in human B cells, B cells counts are much less affected, either in COVID-19 or SAVI syndromes. The recognition of delayed STING over-activation in severe COVID-19 patients could prompt to target STING with specific small molecules inhibitors already designed and/or aspirin, which inhibits cGAS.

## Introduction

To account for the quite different outcomes of coronavirus disease 2019 (COVID-19), including in young people, as well as the lower prognosis of male, obese, and aged patients, we previously put forward the hypothesis that a delayed over-activation of the stimulator of interferon (IFN) genes (STING) pathway, could be central to the pathogenesis of severe COVID-19 (1, 2). This delayed over-activation could be the consequence of gain of function in the cGAS-STING axis, and/or cytosolic damages induced by severe acute respiratory syndrome coronaviruses (SARS-CoV-2) in epithelial, endothelial, or innate cells (1, 2). This hypothesis was partly deduced from the observation that bats withstand SARS-CoV viruses, thanks to a loss of function mutation of STING, associated with higher synthesis of IFN-α and much lower synthesis of IFN-β (3).

This hypothesis would fit with the Kawasaki-like features and high thrombosis rate observed in severe COVID-19 (2). Indeed, over-activation of the STING pathway can lead to the release of IFNβ and tissue factor (through induction of pyroptosis by the STING-gasdermin pathway) in infected epithelial cells and/or endothelium (2).

As functional consequences of DNA sensing by the cGAS-STING pathway differ according to antigen presenting cells or lymphocytes (4), which had not been the focus of previous articles (1, 2), the present review aims: i) to study arguments for a possible contribution of over-activation of the cGAS-STING pathways to the disturbances of T and B cell responses observed in previous SARS-CoV, and in severe COVID-19 (**Table 1**); ii) to raise further hypotheses to test in COVID-19 (**Table 2**).

**Table 1:** Similarities in T and B cells features in severe coronavirus disease 2019 (COVID-19), and STING over-activation, including SAVI (STING-associated vasculopathy with onset in infancy) syndromes.

	Severe COVID-19	STING overactivation
Differentiation at onset	Th2	Th2
T cells phenotype	Exhausted	Exhausted
T cell counts	Marked lymphopenia	Marked lymphopenia
T cell apoptosis	Increased	Increased
Tregs	Reduced number	Reduced number
B cell counts	Declined	Declined
Antibodies	Poorly efficient	Deficiency (in mice)
Myeloid cells	Expansion	Expansion
IL-6 levels	High	High
Pneumonitis	Worsened by T cells	Worsened by T cells

**Table 2:** Questions and hypotheses to address.

Can some subsets of T cells be infected by SARS-CoV-2, following expression of CD147 or CD26, especially activated and exhausted memory T cells? Does SARS-CoV-2 reduce the ratio of full-length wild-type human STING/truncated STING isoforms in antigen presenting cells at early stages of COVID-19? Does SARS-CoV-2 activate aryl hydrocarbon receptor, like α-coronaviruses do? In COVID-19, is IFN-β more detrimental than helpful when given to patients already admitted in ICU units? Do Jak-inhibitors enhance or reduce the replication of SARS-Cov2 *in vitro* and *in vivo* together with the α and β IFNs levels? Is the subdomain within the C terminus domain (CTT) of STING (miniCTT) different in patients with severe COVID-19? Are GM-CSF+ CD4 T cells capable of prodigious ex vivo IL-6 and IFN-γ production in critically ill COVID-19 patients infected by SARS-CoV-2? Is IL-6 negative feedback on cGAS-STING activation abolished in severe SARS-Cov infections by inhibition of ULK1 (and autophagy) by the SARS-CoV viruses? Is this defect increased by concurrent infections by herpes-viruses? Which mechanisms are mostly responsible for the down-regulation of STING activity in T and B cells, as compared to myeloid immune cells and non-immune cells: trafficking, degradation, miRNA-mediated repression, or post-translational modifications? Are Tregs even more prone to exhaustion and/or lymphopenia than effector T cells in mouse or humans with gain of function mutations of STING? Does gain of function and/or activation of some STING-pathways in helper T cells, including Tfh, lead to their premature apoptosis and contribute to the rather short duration of antibodies towards SARS-CoV infections? Is the functionality of some STING pathways impaired in subsets of memory B and T cells in SAVI syndromes and COVID-19? Does concurrent EBV and SARS-CoV-2 infections in B cells increase the exhaustion of T lymphocytes by over-activated presenting B cells? Is miR-576-3p deficient in T cells from severe COVID-19?

### *In Vivo* Infection of T Cells by SARS-CoV-2 Has Not Yet Been Demonstrated

SARS-CoV-2 invades most host cells *via* binding of its structural spike glycoprotein to angiotensin-converting enzyme 2 (ACE2) (5, 6). Although ACE2 is upregulated by type I IFN and IFN-γ, and to a lesser extent type II IFNs (7), but not type III IFN (8), it is usually not expressed in immune cells (5, 6), especially in T and B cells.

Nevertheless, it was shown that some immune cells, including T cells, can be infected by the SARS-CoVs and middle-east respiratory syndrome coronavirus (MERS-CoVs) (9, 10) [although they poorly replicate in lymphocytes (9)]. This suggests that other receptors can contribute to entry of those SARS-CoVs in some lymphocytes. A first possibility could be CD147 [also known as basigin (5, 11)]. CD147 is strongly expressed in whole blood, neutrophils, classical monocytes, macrophages, plasmacytoid dendritic cells, NK cells, naïve CD4+ T cells, terminal effector CD4+ T cells, naïve CD8+ T cells, effector memory CD8+ T cells, naïve B cells, and plasmablasts (5, 12). It has also been suggested that CD147 could act as a secondary receptor for SARS-CoV-2 in T cell lines (10) (**Table 2**).

CD26 (DPP4) is another receptor important in SARS-CoV infections, described in MERS-CoV, and potentially recognizing SARS-CoV-2 (13). Similar to CD147, CD26 is expressed in nearly all immune cells, but, contrary to CD147, not in B cells (5).

**Figure 1:**
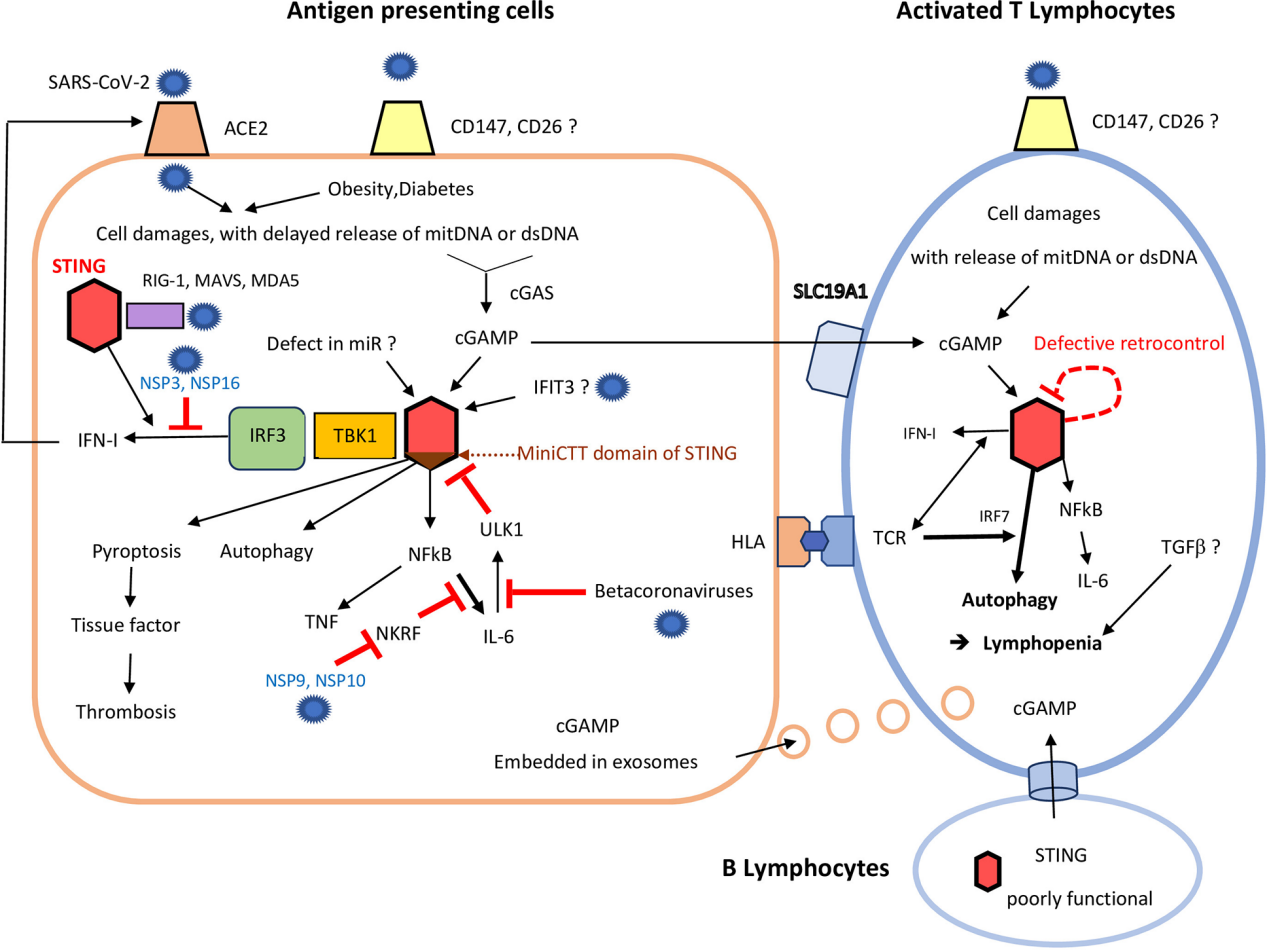
Consequences of STING over-activation on antigen presenting cells and T cells following severe acute respiratory syndrome coronaviruses (SARS-CoV-2) infection. In antigen presenting cells (left), due to poor virus control by RNA sensors (including RIG-1, and MDA5) and despite the help of STING (red hexagon), SARS-CoV-2 induces delayed cell damages, with mitochondrial DNA and dsDNA release. It can add to damaged self-DNA secondary to ageing and/or obesity/diabetes. cGAS catalyzes those self-DNA in cyclic nucleotides, mainly cGAMP, which in turn activates STING (red hexagon). This activation of STING may be enhanced by: i) a lack of miRNAs, (like miR-576-3p, which normally suppress STING translation); ii) excess of IFIT3, which interacts with STING gene to promote its expression. Binding of cGAMP to STING first induces activation of the STING-TBK1-IRF3 pathway, leading to IFN-I synthesis. In COVID-19, the SARS-CoV-2 NSP 3 and 16 lower this IFN secretion, but IFN enhances expression of ACE2 on cell membrane. cGAMP, which can be released to by-stander cells through viral exosomes, can also be transmitted by B cells (low), to activated T cells (right), through specific channels. Independently of the STING-TBK1-IRF3 pathway, binding of cGAMP to STING can also: i) induce pyroptosis; ii) promote autophagy and lymphopenia; iii) activate NF-κB and both TNF and IL-6 synthesis. SARS-CoV-2 NSP 9 and 10 still enhances IL-6 release through inhibition of NKRF. Some beta-coronaviruses can also impede ULK1 and its negative feed-back loop, further enhancing IL-6 secretion. Excess of cGAMP can be internalized by by-stander activated CD4+ and CD8+T cells, which highly express the cGAMP importer SLC19A1. Therefore, even without infection of T lymphocytes, STING activation could also occur in activated T lymphocytes, especially those already stressed by a continuous activation by their hyper-responsive TCR. Defective retro-control of STING (dashed red loop) by IL-6 (see above) could still enhance STING activation in severe COVID-19. In activated T cells, STING overactivation leads to autophagy, up to lymphocyte death. This contributes much to the lymphopenia observed in rodent and human gain of function of STING models, and perhaps also in COVID-19, with subsequent poor virus control.

However, contributions of CD147 and CD26 to COVID-19 still remain unproven, and ACE2 should be still considered as the only receptor for SARS-CoV-2 (14) (**Figure 1**).

### The STING Pathway Modulate Various T Cells Functions

#### STING Is the Major Sensor of Self and Foreign Cytosolic DNA

STING is a pattern recognition receptor localized in the endoplasmic reticulum (ER) membrane. The cGAS-STING pathway plays a central role in sensing cytosolic DNA upon infection with DNA from bacteria and DNA-viruses (including endogenous retroviruses). Lack of expression of STING by some cell types contribute to preferential homing of DNA viruses. For instance, hepatitis B virus has a tropism for human hepatocytes, which have undetectable levels of STING protein (15).

Those DNA viruses can reduce STING signaling by increasing autophagy-mediated turnover of STING or interfering with STING trafficking. They can also interfere with IRF signaling and antiviral IFN I responses, rather than with NF-κB responses. For instance, herpes-virus 1 (HSV-1), reduce the ratio of full-length human STING/truncated STING isoforms, induced by alternative splicing of STING RNAs. The three STING truncated isoforms fail to induce IFN-β, and they act as selective pathway inhibitors of full-length STING, even in combination with upstream inducer cyclic-di-GMP-AMP synthase (16).

Importantly, although mainly a DNA sensor, STING is also necessary for full control of enveloped RNA viruses, like influenza virus and coronaviruses, independently of cGAS. Indeed, sensing of lipid membrane fusion through STING also contributes to the antiviral response against enveloped RNA viruses, so that complete protection against RNA viruses also relies on STING (17). STING can also participate in viral RNA sensing through its interaction with the mitochondrial antiviral signaling protein (MAVS) (17). STING further transduces the signaling induced by RNA-derived PAMPs through retinoic acid-inducible gene (RIG-I)-like sensors (RIG-I and melanoma differentiation-associated protein 5 (MDA-5) (17) (**Figure 1**).

Multiple *Flaviviridae* (including zikaviruses, dengue virus, West Nile virus, Japanese encephalitis virus) and hepatitis C virus disrupt STING-mediated signaling (18). Some do so by cleaving STING or cGAS, but others only interfere with the STING-TBK1 interaction (18), explaining why the IFN axis can be affected but not the NF-κB axis (19).

So far, there is no evidence that betacoronaviruses increase autophagy-mediated turnover of STING, interfere with STING trafficking, or cleave STING or cGAS. However, it has been shown that various SARS-CoV-2 protases [non structural protein 3 (NSP3) and NSP16] strongly inhibit the downstream STING-TBK1-IRF3 pathway and IFN synthesis (20). Conversely, SARS-CoV-2 NSP9 and NSP10 protases still increase signaling and IL-6 production by inhibition of NKRF, an endogenous NF-κB repressor (21) (**Figure 1**).

Importantly, STING also senses intra-cellular damaged self-DNA [mitochondrial DNA, and self-dsDNA released in extra-cellular space, including dsDNA from neutrophil extracellular traps (NETs) (22)] secondary to bacterial or viral infections. Therefore, at late stages of RNA viruses infection, the evasion of STING by RNA viruses can be counterbalanced by its activation following released of damaged self-DNA (**Figure 1**).

Upon recognition of non-self (microbial) DNA or self-DNA, the cyclic-GMP-AMP synthase (cGAS) of the host catalyzes the production of cyclic dinucleotides, like cGAMP (from GMPs and AMPs) (**Figure 1**). The length of DNA, rather than its sequence, determines its ability to generate cGAMP following recognition by cGAS (23). Since the endogenous DNA fragments are often short in size, whereas viral and bacterial DNA fragments are generally much longer, this can partly permit discrimination between self and non-self DNA. However, a large amount of self-DNA in cytoplasm outside of the nucleus or mitochondria can also activate the cGAS-STING pathway.

Once activated, STING recruits TANK-binding-kinase (TBK1), which can activate and phosphorylate IFN-regulatory factor 3 (IRF3), to induce type-I IFNs. Those type I IFN then engage the IFN I receptor, thereby activating the JAK/STAT pathways and inducing the transcription of IFN-stimulated genes (ISGs) (23). As the STING gene is itself an ISG, a positive feedback loop can ensue (24). This STING-TBK1-IRF3 axis can be selectively inhibited by viral protease in COVID-19 (20).

However, STING is also a cytosolic hub for other pathways, which can be activated independently of the IFN-TBK1-IRF3 axis. For instance, downstream signaling of STING also lead to NF-κB and/or inflammasomes activation, and secretion of various cytokines, like IL-6 and TNF (1, 16), as also observed in COVID-19 (21) (**Figure 1**).

STING is highly conserved throughout evolution, being observed >500 million years ago, and has also other primitive functions than secretion of IFN-I and cytokines to fight viruses or intra-cellular bacteria (23, 25, 26).

First, it activates the process of autophagy (critical for the elimination of DNA and viruses in the cytosol of innate immune cells), independently of TBK1 activation and IFN-I signals (25) (**Figure 1**).

Second, if foreign nucleic acids accumulate despite autophagy, further activation of the STING pathway can lead to growth arrest, up to apoptosis or pyroptosis (23), which can contribute to increase the risk of thrombosis (Figure) (2). Those STING-mediated cell deaths might be critical for preventing pathogen dissemination (23, 26).

Reciprocally, some bacterial pathogens (and possibly some DNA viruses) have learned to exploit those STING-induced deaths to impair T cell response (27). They sort their DNAs into extracellular vesicles and deliver them to T cells, to excessively stimulate the cGAS-STING pathway, after ingestion, which primes those T cells for apoptosis (27, 28). In HSV-1 infections, STING can also be directly transferred to previously uninfected cells through exosomes (29) (**Figure 1**) in order to force those cells to detect earlier their invasion by HSV-1.

This strategy should less apply to the SARS-CoVs, since they are RNA viruses, less directly recognized by cGAS-STING. However, SARS-CoVs could indirectly tease T cell response through the cGAS-STING pathway. Indeed, viral-induced DNA damage in by-stander cells of T cells (including B cells) can lead to cGAMP synthesis in those neighbor cells (**Figure 1**). Transfer of this cGAMP to T cells can also activate the cGAS-STING pathway in activated CD4+ and CD8+ T cells. Indeed, those activated T cells highly express the cGAMP importer SLC19A1 (whereas naïve T cells do not) (**Figure 1**) (28).

SLC19A1 is a folate-organic phosphate antiporter, and the major transporter of CDNs like cGAMP through the cell membranes, of activated T cells (30). Interestingly, in human cell lines and primary cells *ex vivo*, CDN uptake through SLC19A1 is inhibited by both folates and two anti-folates medications of rheumatoid arthritis and other inflammatory rheumatisms: sulfasalazine and methotrexate (30). Accordingly, the outcome of rheumatoid arthritis with severe COVID-19 treated or not by methotrexate or sulfasalazine could be worth to study, although methotrexate as only limited efficacy against SAVI (STING-associated vasculopathy with onset in infancy) syndromes.

#### Consequences of cGAS-STING Activation in Murine and Human T Cells

##### STING Is a Co-Signal Which Modulates Many T Cell Function in Mice

STING is strongly expressed in most cells, except human B cells, and recent studies showed that mouse CD4+ T cells express even higher amounts of STING protein than macrophages and dendritic cells (28). The activation of mouse T cells upon T cell receptor (TCR) recognition of antigen peptide-MHC is regulated by STING (23, 31), which can modulate various T cell functions, such as cytokine production, cell growth, and differentiation (23). STING can make the mouse T lymphocytes more responsive to ER stress induced by T cell receptor (TCR) signaling. It also leads to lymphocytes death following excessive triggering of TCR, in cells with other sources of ER stress (32, 33).

##### Some Features of STING Signaling Seem Less Efficient in Human T Cells

In humans, key components of the DNA sensing machinery are expressed in activated T cells, including STING itself (34). However, as compared with other cells (including myeloid immune cell types and non-immune cells), STING seems much less functional in human T cells, at least regarding IFN secretion (**Figure 1**) (18). It has been highlighted in the most recent review on STING that lack of an IFN I response downstream of STING in human T and B lymphocytes is in line with the observation that functional DNA sensing pathway in T and B lymphocytes would lead to continuous IFN I production in those highly proliferative cells and detrimental auto-inflammatory diseases (18).

A compromised expression of the cGAS-STING cascade has also been confirmed in CD8+T cells from cancer patients, with reduced stem-like central memory CD8+T cells subsets (35). This is a selective advantage for survival and growth for cancers (36). Several studies showed that STING re-activation can stimulate antitumor immune responses in numerous cancers, including leukemia, melanoma, glioma, and hepatocellular carcinoma (37).

In HIV patients, resting CD4+ T cells, some of which harbor HIV, are also defective in STING-dependent IFN I production (34). A defective STING pathway may render human T cells vulnerable to other pathogens, due to low production of IFN, including other retroviruses (e.g., human T-lymphotropic virus type 1), or viruses (e.g., VZV and HHV 6) that replicate through a DNA (18). Conversely, other downstream signaling of STING than IFN, like NFκB, are less affected in human T cells, as shown in HIV-1 lymphocytes (38). This is also observed in SAVI syndromes (induced by gain of function of STING), where over-activation of STING by viruses can lead to T cell hyper-responsiveness with IL-6 secretion (as well as T cell exhaustion, and autophagy-induced lymphopenia).

##### Sources of Activation of STING Within T Lymphocytes

The final sources of cGAMP for the activation of STING in T cells should differ according to the possibility (or not) of a direct infection of T cells by SARS-CoV-2 through CD147 or CD26. Such infection has not yet been demonstrated, but might be searched in subsets of activated T cells of severe COVID-19) (**Table 2**), including memory T cells which upon activation exhibit much greater up-regulation of CD147 than naïve T cells (39) (**Figure 1**).

In such event, STING might be activated by: i) cGAMP incorporated into viral particles during encapsidation in antigen presenting cells, to accelerate STING activation in neighboring T cells and their apoptosis; ii) endogenous cGAMP synthetized from damaged cytosolic self-DNA within T cells, including virally damaged mitochondrial self-DNA, as shown for dengue virus infected cells (40) (T cells are permissive for dengue virus); iii) a non-canonical mechanism involving lipid membrane alterations, as observed following influenza virus infection in mouse embryonic fibroblast cells (41).

However, even if T cells are not infected by SARS-CoV-2, STING could still be activated within T cells by: iv) exogenous cGAMP released in extra-cellular spaces by dying cells, and internalized by activated CD4+ and CD8+T cells (15) (which highly express the cGAMP importer SLC19A1); v) cGAMP transmitted by B cells to T cells *via* other cellular channels; vi) DNA damages occurring in T cells themselves, including oxidized mitochondrial-DNA (**Figure 1**).

### T Cell Responses Observed in Coronavirus Disease 2019 and Following STING Activation

#### Th2 Differentiation at Early Stages

##### A Th2 Immune Response Is Observed at Onset of Coronavirus Disease 2019

At 24h after the first clinical signs of COVID-19, interleukins (IL-1α, IL-1β, IL-6, IL-10), and IFNs (IFN-α2, IFN-β1, IFN-II) are significantly elevated, but the cellular sources of those cytokines are probably more epithelial and innate cells than T lymphocytes (42). After several days, naïve lymphocytes differentiate in Th2, in line with the role of eosinophils in fighting RNA viruses [ascribed to the RNAses inside their granules (43)]. Such Th2 profile had previously been described in SARS patients, and lethal outcomes correlated with elevated Th2 cell serum cytokines (44).

##### A Th2 Immune Response Is Also Induced by STING Activation

The main ligand for STING, cGAMP, can also preferentially induce a Th2 differentiation (23, 45). Co-stimulation of antigen presenting cells and T cells by foreign or self-DNA similarly suppresses T-bet expression, followed by the induction of GATA-3 and Th2 cytokines (23, 45). The extra-cellular self dsDNA of neutrophil extracellular traps (NETs) induced by rhinovirus infection of upper airways is associated with a preferential Th2 response (46). Accordingly, the shift towards a Th2 response in early stages of COVID-19 would fit with STING activation (**Table 1**).

However, the expression of the aryl hydrocarbon receptor (AhR) on T cells following CD28 activation can later shift Th1/Th2 balance towards a Th1 response (45). This could explain why, in lungs of mice models of SAVI syndromes, Th1, rather than Th2, seem to play a critical role in tissue damage and the persistence of inflammation (47). Whether SARS-CoV-2 could activate AhR has not been addressed so far (**Table 2**), but a murine coronavirus does it, contributing to cytokine modulation and viral infection (48).

#### Exhausted/Senescent Phenotypes Are Observed in Severe Coronavirus Disease 2019, But Also Following STING Over-Activation

In most patients, COVID-19 is a benign and even asymptomatic infection, and recognition of some viral peptides by T cells (including some memory T cells already primed by previous encounter with human or animal coronaviruses) could help innate cells to clear SARS-CoV2. Recent findings using immunospot essay assessing IFNγ production, showed that 6/8 contacts of index patients developed a SARS-Cov-2 specific T cell response lasting up to 80 days against structural and/or accessory SARS-CoV-2 proteins, although none developed antibodies, and all were tested negative by PCR (49).

Conversely, due to genetically encoded differences in innate and/or T cells, or epigenetic changes acquired during life, innate and/or adaptive cells could remain inefficient to clear SARS-CoV-2 in the minor subset of patients with severe COVID-19. Those genetically encoded differences might be loss of function mutations polymorphisms: inborn errors of TLR3- and IRF7-dependent type I IFN immunity have been found in 3.5% of life-threatening COVID-19 pneumonia in patients with no prior severe infections (50). However, gain of function mutations in other genes, including genes of the STING pathways, should also be searched, since delayed over-reaction to virus damage might be as important as poor initial control of SARS-CoV-2.

##### Exhaustion of T Cell Lymphocytes With Delayed Secretion of IL-6 in Severe Coronavirus Disease 2019

Increased presence of strongly activated T cells, characterized by expression of HLA-DR, CD38, CD69, CD25, CD44, and Ki-67 has been reported in several studies on COVID-19 (51). In intensive care (ICU) units, both virus-specific CD4 and CD8 T cells were detected in all COVID-19 patients (at average frequencies of 1.4 and 1.3%, respectively), with phenotypes suggestive of either CD4 central memory, or CD8 effector memory T cells (52).

Although SARS-CoV-2 restrains antigen presentation by down-regulating MHC class I and II molecules, CD4+ T cells, and even more CD8+ T cells (20, 53), exhibit functional exhaustion/senescent patterns (51). The expression of NK group 2 member A receptor (NKG2A), programmed cell death 1 (PD-1), and T-cell immunoglobulin and mucin containing protein-3 (TIM-3) (51) increased as patients progressed from prodromal to overtly symptomatic stages (54). In critically ill patients, cellular functionality was also shown to be impaired in CD4 and CD8 T cells (20). This could explain why, despite initial over-activation of lymphocytes, at autopsy, patients who succumbed to infection all had high virus levels in the respiratory tract and other tissues (55).

Elevations in CRP (C-reactive protein), strongly associated to interleukin (IL)-6 levels, appear to be unique to COVID-19 patients when compared to other viral infections (20). Those elevated IL-6 levels are associated with ICU admission, respiratory failure, and poor prognosis (20). Various activated and expanded antigen presenting cells, including macrophages and dendritic cells, seem usually responsible for the cytokine storm with high IL-6 secretion, and for the high serum levels of CRP (54). This is in line with previous studies on other betacoronaviruses human infections (SARS-CoV and MERS-CoV). Indeed, most of the exhausted T cells express less cytokines than innate cells, and T cell numbers are negatively correlated to serum IL-6, IL-10, and TNF-α concentration. However, IL-6 increases fairly late during the disease’s course |20] [which much reduces its prognostic value at earlier stages (56)].

A first hypothesis for this delayed raise of IL-6 could be the deferred re-expression of ACE2 receptor (57) on previously infected epithelial cells, following IFN secretion, leading to re-activation of macrophages and dendritic cells (**Figure 1**). A second hypothesis is an increased peripheral blood frequency of a subset of polyclonal GM-CSF+ CD4 T cells capable of prodigious *ex-vivo* IL-6 and IFN-γ production, which has been described in COVID-19, although only in critically ill patients (58).

##### Over-Activation of the STING-Pathway Can Also Activate T Lymphocytes, and Lead to Secretion of IL-6

Unlike in innate immune cells, cGAMP alone does not induce IFN-I production by mouse naïve CD4+ and CD8+ T cells, since both TCR stimulation and STING activation are required (23). Conversely, in effector CD4+ and CD8+ T cells, the STING activation by cGAMP can lead to IFN-I and probably various cytokines production, even without TCR co-stimulation (although cGAMP-induced IFN-I production is further increased by IL-2 and TCR binding) (23). The amount of IFN-I produced by mouse effector Th1 and activated CD8+ T cells in response to co-stimulation with TCR and cGAMP is even higher than in dendritic cells (23). This is in line with the much greater ability of previously activated T cells to internalize cGAMP from neighbor cells, as compared to naïve T cells (22).

This continuous secretion of IFNs by effector T cells help to clear intra-cellular pathogens at the early stages of viral infections. Early or preventive treatment by IFN-α or IFN-β improved the outcomes of SARS-CoV and MERS-CoV infection in mice and in non-human primates. However, some viruses, including SARS-CoVs, not only can lower IFN production, but can also take advantage of some subtypes of IFN, like IFN-β, to persist in the host at later stages (7). This could explain why in humans, survival rates in MERS-CoV infection were not increased, possibly because those drugs were given too late (59). At this stage, the delayed IFN-β response in some murine models of severe acute respiratory syndrome (SARS) is, on the opposite, associated with excessive influx of pathogenic inflammatory monocytes-macrophages and a much worse prognosis (60) (**Table 2**).

STING over-activation might similarly paradoxically enhance SARS-CoV-2 replication in lung monocytes-macrophages through transient increase secretion of IFN-β. This contributes to T lymphocyte exhaustion up to final fall of IFN-I secretion. Indeed, following infection by gamma-herpesvirus 68 (γHV68), the increased secretion of IFN-I also promoted the replication of this virus in cultured macrophages of a mouse model of gain of function of STING (N153S) (61). Furthermore, IFN-β can also induce up-regulation of ACE2 in airway epithelia (7) (**Figure 1**). This might fuel new infections by SARS-CoV2, and impair antigen-specific T cell responses to SARS-CoV antigens (60).

The contribution of IFN-β to COVID-19 remains quite uncertain however in humans, since: i) IFN-I over-secretion induced early by STING activation in infected cells is quickly counterbalanced by a strong inhibition of the downstream STING-TBK1-IRF3 pathway by various SARS-CoV-2 protases (**Figure 1**) (20); ii) IFN-I, II and III expression was seemingly not significantly increased in lung tissues infected with SARS-CoV-2 (62); iii) although an increased expression of IFN-I, and IFN-I related genes, were observed in human blood during the first stages of COVID-19, they declined when the patient got worse (63).

Nevertheless, STING activation can lead to nuclear-factor kappa B (NFκB) and cytokine secretion, including IL-6, independently of IFN secretion. First, SARS-CoV-2 non-structural protein (NSP)9 and NSP10 still increase IL-6 production, by inhibition of NF-κB-repressing factor (NKRF), an endogenous NF-κB repressor (21) (**Figure 1**). Second, whereas the induction of IFN-response genes are maximally up-regulated in PBMCs from SAVI patients (exposure to cGAMP brought about no change), conversely, transcription of the genes encoding tumor necrosis factor (TNF) and IL-6 was elevated in unstimulated peripheral blood mononuclear cells (PBMCs) from SAVI patients, and was still augmented on exposure to cGAMP (64). This is reminiscent of what is observed in severe COVID-19 patients (**Table 1**) (65).

A subdomain within the C terminus domain (CTT) of STING (mini_CTT), appears to be required for STING-mediated, TBK1/IRF3-independent, NF-κB activation in a human system (31). This mini_CTT domain might also be required for cGAMP-induced inhibition of mammalian target of rapamycin complex 1 (mTORC1) signals in T cells (**Figure 1**). Accordingly, gain of function of this subdomain of STING could be preferentially studied in young patients with severe COVID-19 (**Table 2**).

#### Lymphopenia Is an Early Prognosis Factor in Coronavirus 2019, and Is Also Induced by STING Over-Activation

##### T Cell Lymphopenia and Severe Coronavirus 2019

In COVID-19, a sustained decrease in CD4+ and CD8+ T cells, especially CD8^+^ T cells, is observed, despite an increase in IL-6, IL-10, IL-2, IFN-γ levels, and neutrophil counts. This fall is even more pronounced in patients requiring ICU (54), whatever the age (42). Decreased counts of NK cells, eosinophils (53), and γδ-T cells (66), are also observed in severe cases (53). In most severe COVID-19, this lymphopenia is associated with atrophy of lymphoid organs (67). Regulatory T cells are moderately increased at the onset of mild COVID-19, but similarly later decline in severe cases (68).

In mild cases, this lymphopenia might partly reflect redistribution of lymphocytes to lymphoid organs and tissues, since IFN-I, IL-6, and TNF-α can promote retention of activated lymphocyte in lymphoid organs (54). However, although some autopsy study showed extensive lymphocyte infiltration in the lungs (55), others observed only found neutrophilic infiltration in the lung (69), or death of lymphocytes in spleen and lymph nodes (70).

This T cell lymphopenia appears to predict morbidity and mortality, even at early stages, while elevated levels of CRP, LDH, D-dimer, as well as decreased blood platelets are only late prognosis factors (20).Therefore, rather than a cytokine storm induced by NF-κB stimulation, a marked and widespread virus-induced lymphopenia may be the real cause of death in many patients (71).

##### Excess of TGF-Signaling Could Contribute to This Lymphopenia

Among the numerous mechanisms for the functional exhaustion/senescent patterns of T lymphocytes in severe COVID-19, the release of TGF-β1 could contribute to impair T cell function (and may also be responsible for switch in IgG to IgA production observed in COVID-19 patients). TGF-β1 levels are indeed associated with more severe COVID-19 (72). Similar observations were previously made in patients with severe acute respiratory syndrome (SARS): TGF-β1 was continuously up-regulated during the entirety of SARS, and its prolonged over-production was associated with severity of SARS and memory TCD8 depletion (73). Whether STING also increase TGF-β1 signaling has not been directly addressed in humans, but in rodents STING deficiency in liver effectively reduced the severity of hepatic fibrosis, which was closely associated with the inhibition of TGF-β1 signaling (74). Therefore, STING activation could also reinforce some TGF-β functions in humans (75) and contribute to lymphopenia (**Figure 1**).

##### But T Cell Lymphopenia Is Also a Direct Consequence of STING Over-Activation

In humans, a marked lymphopenia with reduced memory CD4+ and CD8+ T cells in the periphery (23) is a major feature of SAVI syndrome (76). In a knock-in model carrying an amino acid substitution (V154M) in mouse STING (corresponding to a mutation seen in human patients), the mice also developed a severe combined immunodeficiency disease affecting B, T, and NK cells, with a significant compensatory expansion of monocytes and granulocytes (77). This phenotype is reminiscent of what is observed in severe COVID-19 (**Table 1**).

B- and T-cell developments were blocked since early immature stages, either in bone marrow or thymus (77). Excess of IFN-I (31) does not contribute to this lymphopenia. Signs of inflammation in lungs and kidneys were also IFN-independent (77), and probably driven by over-reactivity of the TCR (32, 78). This is in line with the observation that in mice STING-associated vasculopathy, STING regulates T cell proliferation in cell culture, independently of IRF3 (32).

Radiations chimeras confirmed that T cell lymphopenia depends on T cell intrinsic expression of the SAVI mutation (79). Co-stimulation of CD4+ T cells by STING and TCR activation can induce growth arrest and lymphopenia by inhibiting mammalian target of rapamycin complex 1 (mTORC1) activation (**Table 1**). This STING-mediated inhibition of mTORC1 signals following TCR activation is partly dependent on both IFN regulatory factor 3 (IRF3) and IFN regulatory factor 7 (IRF7) (**Figure 1**), but not on TBK1 and IKKϵ (23). The identification of this unique pathway in T cells is critical for the development of new therapeutic strategies for targeting STING in T cells, and to prevent lymphopenia (23).

STING mutants also induce chronic activation of ER stress and unfolded protein response (UPR) within T lymphocytes. STING-N154S disrupts calcium homeostasis in T cells, and primes them to become hyper-responsive to T cell receptor signaling induced ER stress, leading to cell death, both in CD4+ and CD8+ T cells (32, 78). The mouse CD4+ and CD8+ T cell death through ER stress can be restored (as well as lung disease) by changing TCR specificity (36).

cGAMP-induced STING activation can also lead to the inhibition of IL-2 signaling pathways, which decreases the synthesis of regulatory T cells (Tregs) (23) (**Table 1**).

##### This T Cell Lymphopenia Can Be Partly Reversed by IL-7

Interleukin 7 (IL-7) is essential for lymphocyte survival and expansion (80). IL-7 therapy has been shown to restore lymphocyte counts and functional activity, leading to decreased viral load and clinical improvement in several life-threatening viral infections (71). The effect of compassionate use of IL-7 in 12 critically ill patients with COVID-19 and severe lymphopenia was compared to the outcome of 13 matched controls who did not benefited from IL-7. IL-7 was associated with a restored lymphocyte count, with the IL-7 group having levels more than two-fold greater than the control group (71). However, functional defects of the exhausted lymphocytes could persist, since at day 30, mortality was 42 and 46%, respectively.

### The Human STING-Associated Vasculopathy With Onset in Infancy Syndromes, Induced by Gain of Function Mutation of STING, Share Other Features Than T Cell Exhaustion and Lymphopenia, With Severe Coronavirus 2019, Including Pneumonitis

SAVI syndromes share other striking similarities with COVID-19: a variable combination of fever, rashes, an inflammatory vasculopathy mimicking lupus chilblains, up to vaso-occlusive process and acral necrosis, and pulmonary inflammation leading to interstitial lung disease (64).

In STING N153S SAVI-like mice model, mice lacking adaptive immunity had no lung disease, and T-cell receptor β chain (Tcrb)^−/−^ STING N153S animals only had mild disease (79). Therefore, T cell over-response seems important for lung disease induction, despite the concurrent lymphopenia induced by STING over-activation (79). Crossing those mice to animals lacking cGAS, IRF3/IRF7, IFN-alpha/beta receptor alpha chain (IFNAR1), adaptive immunity, αβ T cells, and mature B cells, showed that lung disease developed independently of cGAS, IRF3/IRF7, and IFNAR1, suggesting that other triggers than cyclic dinucleotides (the ligands of cGAS) and/or IFN, contribute to STING over-activation within T cells (79). A defective retro-control of STING (**Figure 1**) might be one explanation for those findings, either in patients with gain of function of STING, and/or severe COVID-19.

In normal cells, IL-6 is a negative feed-back regulator of STING induced by double-stranded DNA, since IL-6 promotes STING degradation by activating/dephosphorylating UNC-51-like kinase (ULK1) (65) (**Figure 1**). It would be worth testing the hypothesis that this feedback is mitigated in severe COVID-19 by modulation of autophagy (81), and inhibition of molecules like ULK1 by SARS-CoVs (**Table 2**), in as much they can infect activated/memory T cells expressing CD147. Indeed, HSV-1 can inhibit ULK1 to escape the autophagy process (82), and previous studies on other betacoronaviruses showed that, upon cell infection, these viruses inhibit macro-autophagy (83). For instance, the porcine hemagglutinating encephalomyelitis (PHEV) betacoronavirus inhibits the expression of the ULK1 protein (84). Such virally induced lack of negative feedback on STING expression might indeed contribute, together with gain of function variants of the cGAS-STING pathway variants, to a delayed increase of STING activation in activated CD4+ or CD8+ memory T cells following infection by betacoronaviruses, and worse pneumonitis.

The paradoxical poor control of virus in lung macrophages of SAVI models is partly due to excess of IFN-I and II, as deduced by a strong increase of expression of IFN-stimulated genes, which leads to ACE2 over-expression (**Figure 1**). The over-response of some T cell subsets to viral antigens in the lung of SAVI and COVID-19, with subsequent fibrosis (61), could also be the consequence of an imbalance between effector T cells and Tregs. Normally, cytosolic DNA sensing *via* the STING/IFN-β pathway also induces indoleamine 2,3 dioxygenase (IDO). IDO then catabolizes tryptophan to suppress effector and helper T-cell responses and activate Foxp3-lineage CD4(+) Tregs (85). However, Tregs might be even more prone to exhaustion and/or lymphopenia than effector T cells in mouse or humans with over-activation of cGAS-STING, since marked decreases of Tregs have been reported in severe COVID-19 (51) (**Table 2**).

### B Cell Responses

#### B Cell Responses in Coronavirus Disease 2019

Like T cells counts, blood B cells count decline with COVID-19 severity [lower counts being associated with increased length of virus shedding (86)], albeit B cell lymphopenia is much less pronounced than T cell lymphopenia (25, 87).

Like T cell, B cells are markedly activated, with highly oligoclonal B cell populations, and profound CD27+CD38+ plasmablasts expansion in some patients. This plasmablast expansion is uncorrelated with decreases in memory B cell subsets, or with the limited changes observed in the circulating T follicular helper cells (cTfh) compartment (87). Long-term studies on serology kinetics in SARS-CoV and MERS-CoV infections, showed that most IgG were neutralizing but waned over time, and usually in less than 3 years, with longer durations of detectable antibody associated with more severe symptoms (88). Whether gain of function and/or activation of STING in subsets of cTfh cells leading to their premature apoptosis due to hyperresponsiveness to TCR signals, contribute to this rather short duration of antibodies towards SARS-CoV infections has not been addressed so far (**Table 2**).

Sero-conversion occurs between 7 and 14 days after the onset, but this robust antibody response alone is insufficient to avoid severe COVID-19 (20). The high levels of anti-SARS-CoV-2 antibodies in aged COVID-19 do not prevent from severe clinical outcomes, and COVID-19 ICU patients often have high titers SARS-CoV-2 specific antibodies (89).

Another argument suggesting that antibodies might be less important than T cells to control SARS-CoV-2 is the possibility of only mild COVID-19 in patients with severe hypogammaglobulinemia. In a report on seven patients (32 to 79 years-old) with primary antibody deficiencies and COVID-19, five had common variable immune deficiencies (CVIDs) (dysfunctional B lymphocytes), and two had agammaglobulinemia (lacking B lymphocytes). All were substituted and had similar immunoglobulins levels. Whereas the two patients with agammaglobulinemia had a benign COVID-19 course, the five patients with CVIDs presented with a severe form of COVID-19, requiring treatment with multiple drugs, including IL-6–blocking drugs and mechanical ventilation (90). This suggests an active contribution of over-activated but pathogenic subsets of T-bet+ B cells in severe COVID-19 (87), in line with the observation that the granulomatous-lymphocytic interstitial lung diseases occurring in 10% of patients with CVID are partially reversed by B-cell–depleting drugs (90).

#### B Cell Responses and STING

##### In Mice

B cell receptor (BCR) and STING signaling pathways act synergistically to promote antibodies responses independent of type I IFN (91). However, STING functions autonomously in B cells responding to CDNs, and can be activated by cGAMP without the need of previous BCR ligation (91). This can lead to IFN-β production (92), while IFN-I expression by B cells induces an altered polarization of macrophages toward a regulatory/anti-inflammatory profile, at least *in vitro*, that might benefit to some pathogens (93).

Mitochondria-mediated apoptosis induced by STING is also more pronounced in normal mouse B cells than in other cells, since upon stimulation, STING is degraded less efficiently in B cells (94).

##### In Humans

Activation of MHC class II in human B cells is associated with enhancement of STING signaling (95). However, whereas STING is strongly expressed in mice B cells, in resting humans B cells, STING is poorly expressed and dysfunctional (**Figure 1**), despite the detection of DNA sensing and signaling proteins of the STING pathway [cGAS, gamma-IFN-inducible protein (IFI16), TBK1 and IRF3] (96). The very poor expression of STING in human SAVI B cells could explain why in humans SAVI severe B lymphopenia and hypo-gammaglobulinemia are not observed [whereas in knock-in mice poor B cell development and an almost complete lack of antibodies are found (77)].

This lack of STING signaling in human B cells might be an explanation for the use of B cells as a reservoir for persistent infection by Epstein-Barr virus (EBV), and other human gamma-herpesviruses (96). Consequently, although human B cells appear equipped to sense invading DNA viruses by other sensors than cGAS-STING, yet they fail to induce an IFN-I response upon cytoplasmic DNA exposure in EBV-negative B cells. EBV-transformed B cell lines do express STING, but these lines, as well as STING-reconstituted EBV-negative B cells, do not produce more IFN-I upon dsDNA or cGAMP stimulation than EBV-negative lines, showing that the cytoplasmic cGAS-STING pathway remains dysfunctional, even in EBV-positive human B cells (96).

Of note, a similar abrogation of signal transduction downstream of STING phenomenon has been reported for some subsets of activated human T lymphocytes that produce substantial levels of STING protein (34), and it might also be worth to study the functionality of the STING pathway in all subsets of memory B and T cells in COVID-19 (**Table 2**).

Although they poorly express STING, B lymphocytes could indirectly contribute to activate STING in other T cells in COVID-19. Indeed, to alert other cells of viral infections, triggering of cGAS in infected B cells can result in cGAMP production and packaging, and subsequent transfer of this danger message to other cells, including activated T cells (97) (**Figure 1**).

Consequently, B cells infected by SARS-CoV might contribute to T cell activation and exhaustion in COVID-19, and possibly more frequently in EBV positive patients, which could also contribute to explain why young children have usually much less severe COVID-19 than adults (**Table 2**).

## Discussion: Scenario For T Cells and B Cells Response in Covid-2019

The striking similarities between clinical and biological features of SAVI syndromes and severe COVID-19, as well as other studies on the role of STING on T and B lymphocytes (**Table 1**), support the hypothesis of some delayed over-signaling downstream of cGAS-STING in severe COVID-19, despite initial inhibition of the STING-TBK1-IRF3 axis (and IFN secretion) by papain-like-proteases contained within the NSP3 and NSP16 proteins (98) (**Figure 1**).

A lower IFNα/IFN-β ratio downstream of STING could also promote the replication of SARS-CoV, including SARS-CoV-2, as observed in mice models of SARS-CoV and MERS-CoV (60, 99). Parallel activation of macrophages, dendritic cells, and B cell up to exhaustion, might also lead to excessive triggering of TCR and STING from CD4+ and CD8+ and NK cells, leading to their exhaustion and deaths, and further spreading of SARS-CoV-2.

Cases of severe COVID-19 in very young children suggest that some genetically encoded, and perhaps not yet described, gain of function mutations of the cGAS-STING pathway could be a first explanation for severe SARS-CoV-2 infections in the young. However, most old patients with severe COVID-19 never exhibited previous features of SAVI syndromes, so that other explanations than mutations of cGAS-STING must be discussed, which might also be more specific for SARS-CoV infections.

The first one is mutants in various other molecules controlling the STING pathways. For instance, the Ca2+ sensor stromal interaction molecule 1 (STIM1) ensures correct localization of STING at the ER, and its down-regulation should enhance STING activation (100). A more attractive candidate is IFN induced protein with tetratricopeptide repeats 3 (IFIT3) (**Figure 1**), which is highly up-regulated in SARS-CoV-2 infected bronchial epithelial cells (101): IFIT3 interacts with both STING and TANK-binding kinase 1 genes, and activate them in some disorders like lupus (102).

The second explanation could be poor control of STING expression levels by microRNAs (miRNAs) (**Figure 1**). Induction of miR-576-3p (only present in higher primates: humans, chimpanzee, bonobos, gorilla, and orangutan) by IRF3 and IFN-β triggers a feedback mechanism to suppress STING translation and reduce IFN expression (103). During RNA and DNA virus infections, miR-576-3p sets an antiviral response threshold to likely avoid excessive inflammation (103). Deficient production of miR-576-3p has not yet been searched in severe COVID-19 (**Table 2**).

Therefore, the third and most attractive hypothesis so far is a delayed over-activation of the cGAS-STING pathway, due to a rebound effect following initial STING inhibition by viral proteases. This could occur when damaged self-DNA and mitochondrial DNA [especially in elderly patients, or patients with metabolic disorders (1, 104)], combine with STING over-activation induced by transfer of cGAMP and/or STING from infected antigen presenting cells to T cells (**Table 2**) (**Figure 1**).

This rebound effect might be even more severe in patients with mitigated negative feed-back of STING activation by IL-6, induced by disturbances of the autophagy processes, secondary to ULK1 inhibition by beta-coronaviruses, especially in T cells (65, 81) (**Figure 1**). Lymphopenia, a predictor of poor prognostic, is reversed in 1 week by tocilizumab injection (105). The T CD4+, T CD8+, and NK cells reduced anti-viral cytokine production capabilities and cytotoxic potential, are also partially restored by inhibition of IL-6 by tocilizumab (53). However, inhibition of IL-6 has not yet demonstrated its ability to reduce the death rate in severe COVID-19, although it was associated with seemingly better outcomes of COVID-19 in patients who survived (106). A defect of the negative feed-back of STING activation by IL-6 in most severe cases might contribute to explain those observations.

Confirmation of a delayed over-activation of the STING pathways in severe COVID-19 would prompt to test drugs already designed to specifically and timely control STING activation (107), like endogenous nitro-conjugated linoleic acid (NO2-cLA) (108). Although a reduced release of IFN-I has already been confirmed, those drugs should be tested first in animal models of STING gain of function, to make sure that they also correct STING-induced lymphopenia and NFκB over-activation (108). They may be added to vitamin-D and aspirin, which probably also prevent STING over-activation (2).

STING is indeed a “hub” of the immune response, not restricted to the TBK1-IRF3 pathway, and a driver of cell death, including T lymphocytes death. Directly targeting STING might be more efficient to restrain the delayed cytokine storm and prevent lymphopenia, pneumonitis, and vasculopathy, than blocking only a single cytokine like IL-6. Direct inhibition of STING delayed over-activation in severe COVID-19 might also better protect from premature apoptosis of memory central T cells, and early (109), or late recurrences of SARS-CoV-2 infections, even in young patients.

The striking similarities noticed between SAVI syndromes and signs of severe COVID-19 including pneumonitis (1), inflammatory vasculopathies with acral thrombosis or Kawasaki-like features (2), and the lymphocyte changes described above, are not evidences that the cGAS-STING axis plays a central role in COVID-19 pathogenesis. However, those numerous analogies could prompt to study the contribution of STING activation in T and B cells changes observed during severe COVID-19, and to address some of the questions listed in **Table 2**.

This could reinforce the rationale of using drugs preventing from over-activation of STING to treat COVID-19, including as cheap and well tolerated drugs as vitamin-D and aspirin (2). Interestingly, in a large retrospective study performed in American ICUs, even low-dose aspirin seemed to decrease by half mechanical ventilation, ICU admission, and in-hospital mortality in hospitalized patients with COVID-19 (110). If confirmed by prospective studies, this finding would have major consequences on the future of the COVID-19 pandemic.

## Data Availability Statement

The original contributions presented in the study are included in the article/supplementary material. Further inquiries can be directed to the corresponding author.

## Author Contributions

J-MB and JS had the original idea, and J-MB wrote the first draft of the article. JS, YM, and FL extensively corrected it and suggested new references. All authors contributed to the article and approved the submitted version.

## Conflict of Interest

The authors declare that the research was conducted in the absence of any commercial or financial relationships that could be construed as a potential conflict of interest.

## Abbreviations

ACE2, angiotensin-converting enzyme 2; AhR, aryl hydrocarbon receptor; BCR, B cell receptor; cGAS, protein cyclic GMP-AMP synthase; CIVD, common variable immune deficiencies; cTfh, circulating T follicular helper cells; COVID-19, coronavirus disease 2019; EBV, Epstein-Barr virus; ER, endoplasmic reticulum; GATA-3, GATA3 binding protein; ICU, intensive care units; IDO, indoleamine 2,3 dioxygenase; IFIT3, IFN induced protein with tetratricopeptide repeats 3; IFN, interferon; ISG, IFN-stimulated genes; MAVS, mitochondrial antiviral signaling protein; MDA-5, melanoma differentiation-associated protein 5; MERS-CoV, middle-east respiratory syndrome coronavirus; mTORC1, mammalian target of rapamycin complex 1; NETs, neutrophil extracellular traps; NFκB, nuclear factor-kappa B; NKG2A, NK group 2 member A receptor; NKRF, NF-κB-repressing factor; nsp, non-structural proteins; PAMPs, pathogens-associated molecular patterns; PD-1, programmed cell death 1; PHEV, porcine hemagglutinating encephamomyelitis virus; RIG-1, retinoic acid-inducible gene; SARS-CoV, severe acute respiratory syndrome coronaviruses; SAVI syndrome, STING-associated vasculopathy with onset in infancy; STIM1, Ca2+ sensor stromal interaction molecule 1; STING, stimulator of interferon genes; TBK1, TANK-binding kinase 1; TCR, T cell receptor; TIM-3, T-cell immunoglobulin and mucin containing protein-3; ULK1, Unc-51 like autophagy activating kinase 1; UPR, unfolded protein response.
